# Parental Health Literacy as a Contextual Factor in Proxy-Reported Child Mental Health: A Population-Based Study of Children Aged 6–10 Years

**DOI:** 10.3390/children13020253

**Published:** 2026-02-11

**Authors:** Christian J. Wiedermann, Verena Barbieri, Hendrik Reismann, Giuliano Piccoliori, Doris Hager von Strobele Prainsack

**Affiliations:** 1 Institute of General Practice and Public Health, Claudiana College of Health Professions, 39100 Bolzano, Italy; 2 Faculty of Social Work, Health and Nursing, Ravensburg-Weingarten University of Applied Sciences (RWU), 88250 Weingarten, Germany

**Keywords:** parental health literacy, proxy reports, child mental health, psychosocial outcomes, informant bias, population-based study

## Abstract

**Highlights:**

**What are the main findings?**

- Parental health literacy is consistently associated with proxy-reported mental health outcomes across diagnostic domains in children aged 6–10 years.
- These associations were independent of socioeconomic factors and language context, suggesting added diagnostic relevance beyond background characteristics.

**What are the implications of the main findings?**

- Parental health literacy may shape symptom recognition and reporting, affecting the interpretation of proxy-based mental health screening and diagnostic assessment.
- Considering parental health literacy in early assessments may improve diagnostic accuracy and targeting of follow-up care in child mental health services.

**Abstract:**

**Background/Objectives**: Parental health literacy is linked to child health outcomes, but the evidence relies mainly on parent proxy reports. This study examined the association between parental health literacy and proxy-reported mental health outcomes in children aged 6–10 years and assessed whether these associations reflect general reporting patterns. **Methods**: This study is a secondary analysis of data derived from a population-based cross-sectional survey conducted in South Tyrol, Italy, including proxy data from 3183 children aged 6–10 years. Parental health literacy was categorized as inadequate, problematic, adequate, or missing/insufficient. The outcomes included emotional and behavioral difficulties, psychosomatic complaints, and perceived social support. Linear regression models were estimated for each outcome, adjusted for children’s age, gender, parental age, education, family affluence, migration background, residential setting, and questionnaire language. Selective missingness and insufficient completion of parental health literacy data were examined using logistic regression analysis. Sensitivity analyses were used to adjust the mental health models for social support. **Results**: Higher parental health literacy was associated with lower emotional and behavioral difficulties (B = −1.40, 95% confidence interval [CI] −1.79 to −1.01), higher psychosomatic complaint scores (B = 0.61, 95% CI 0.40 to 0.081), and higher perceived social support (B = 0.14, 95% CI 0.02 to 0.26). The effect sizes were small. Missing or insufficient parental health literacy data showed social patterns by parental education and age, whereas no systematic predictors of early disengagement were observed among parents who partially completed the health literacy instrument. Sensitivity analyses attenuated but did not eliminate the associations between parental health literacy and child mental health outcomes. **Conclusions**: Parental health literacy is associated with proxy-reported psychosocial outcomes in children aged 6–10 years. The consistency of the effects across outcomes suggests that parental health literacy may influence how parents report child functioning, underscoring the importance of considering informant characteristics in proxy-based research.

## 1. Introduction

Mental health problems in children and adolescents are a major public health concern worldwide. Studies conducted before the COVID-19 pandemic consistently estimated that 10–20% of young people experience clinically relevant emotional or behavioral difficulties, showing social gradients and long-term consequences for health, education, and social participation [1,2,3,4]. Since the COVID-19 pandemic, systematic reviews and longitudinal studies have documented a substantial increase in mental health symptoms among children and adolescents, particularly anxiety, depression, and sleep problems [5,6]. In younger children, mental health surveillance relies on parental proxy reports, as self-report instruments are considered developmentally inappropriate before early adolescence [7,8]. Proxy-based instruments, such as the Strengths and Difficulties Questionnaire (SDQ), are widely used in epidemiological studies and public health monitoring [9,10]. Although proxy reporting enables large-scale data collection, it introduces methodological challenges. Evidence shows that parental proxy reports reflect parents’ perceptions, interpretations, and evaluative frameworks, which vary across social, psychological, and cognitive contexts [11,12].

Health literacy (HL), defined as the ability to access, understand, appraise, and apply health information in everyday life [13], may be particularly relevant to this issue. In adults, HL correlates with educational attainment, socioeconomic status, migration background and age [14,15]. Lower HL is associated with poorer health outcomes, less effective health service use, and difficulties in navigating health information environments [16,17]. In child health research, parental HL may influence health-related behaviors, decision-making, and how parents comprehend surveys, interpret symptoms, and evaluate their children’s psychosocial functioning [18].

Previous studies have shown associations between parental HL and child health outcomes, including health behaviors, preventive care, and developmental indicators [16,17]. However, when parental HL and child mental health outcomes are reported by the same informant, associations may reflect informant effects or common method variance rather than child-specific mechanisms [19,20]. Evidence suggests that lower parental HL may be associated with more negative appraisals of children’s functioning, while higher HL may enable more differentiated and optimistic reporting [12,21]. Distinguishing between these possibilities is crucial for interpreting proxy-based findings, particularly during early childhood.

A methodological challenge is the missing data in parental HL assessments. Self-administered HL questionnaires require sustained attention and comprehension, and non-completion occurs more frequently among parents with lower education, socioeconomic resources, or limited HL [13,15,22]. If missing HL data are socially patterned rather than random, complete case analyses may overestimate parental HL and bias associations with child outcomes [23,24]. Despite its importance, selective missingness in parental HL has rarely been examined in mental health research on children.

This study addresses methodological issues using data from a population-based survey in Northern Italy’s bilingual region. Focusing on children aged 6–10 years, where mental health outcomes are available only via parental proxy reports, we examined associations between parental HL and proxy-reported child mental health outcomes, including emotional and behavioral difficulties and psychosomatic complaints. Rather than interpreting these as causal effects on child mental health, we adopt a methodological perspective, asking whether the observed patterns are consistent with informant effects and selective non-response.

**Figure 1:**
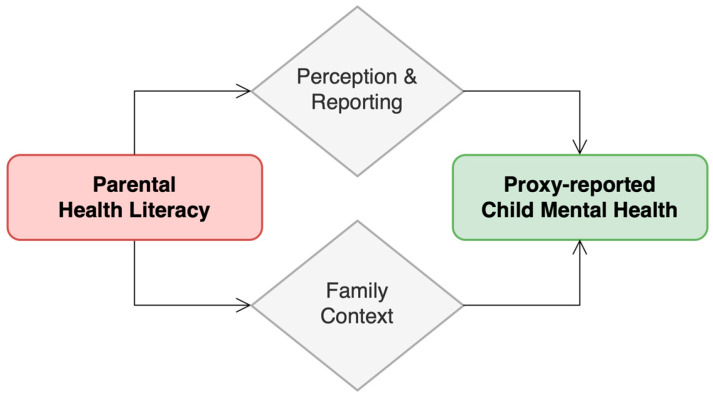
Parental health literacy and proxy-reported psychosocial outcomes in childhood. The diagram highlights that parental health literacy may influence both the child’s psychosocial environment and how parents perceive, interpret, and report emotional and behavioral difficulties, psychosomatic complaints, and perceived social support.

Figure 1 summarizes a conceptual framework developed by the authors, informed by prior empirical research on parental HL, proxy reporting, and informant effects, and illustrates how parental HL may be linked to proxy-reported child psychosocial outcomes through family context as well as perception and reporting processes.

This study aimed to (1) assess the associations between parental HL and multiple proxy-reported child mental health and psychosocial outcomes; (2) examine whether these associations show limited outcome specificity, consistent with global reporting tendencies; and (3) analyze the extent and social patterning of missing parental HL data and its implications.

This study seeks to contribute to the understanding of how parental characteristics, including HL, may shape proxy-based assessments in child mental health research and inform the design and interpretation of future population-based studies involving younger children.

## 2. Methods

### 2.1. Study Design and Participants

This study is a cross-sectional secondary analysis of data drawn from the fourth wave of an ongoing population-based online survey, conducted between 17 March and 13 April 2025 in South Tyrol, a bilingual region of Northern Italy. The survey was administered using the SoSci Survey platform (version 3.2.46, Munich, Germany). Recruitment was conducted through provincial schools. Parents were contacted via email and invited to complete an anonymous, online questionnaire. A reminder email was sent after two weeks of inactivity.

Participation required informed consent, which was obtained electronically before accessing the survey. More than 40,000 families were invited to participate. The survey targeted children and adolescents aged 6–19 years. This analysis focused on children aged 6–10 years, with information obtained via parental proxy reports. This age range was selected because children aged 6–10 years rely exclusively on parental proxy reports in the survey, allowing a methodologically homogeneous assessment of proxy-reported outcomes and informant-related effects of parental health literacy. Depending on the availability of outcome-specific data, the analyses included approximately 2000–2600 children.

All instruments were administered in their validated German and Italian versions. The SDQ was used under a license obtained from the copyright holder, whereas all other instruments were freely available for use in scientific research.

The study protocol was approved by the ethics committee and conducted according to the Declaration of Helsinki principles.

### 2.2. Assessment of Parental Health Literacy

Parental HL was assessed using the European Health Literacy Survey Questionnaire (HLS-EU-Q16) [25,26,27,28]. This instrument measures the difficulty in accessing, understanding, appraising, and applying health-related information across healthcare, disease prevention, and health promotion contexts. Response options ranged from “very difficult” to “very easy.” Following standard scoring procedures [29], responses were dichotomized and summed to yield scores ranging from 0 to 16, with higher values indicating higher HL. The scores were categorized as inadequate (0–8), problematic (9–12), or adequate (13–16). Incomplete HL data were retained as a separate category, defined as fewer than 15 answered items (<15 of 16). Cases with at least one valid response were retained for the analyses of missingness patterns, while score-based analyses were restricted to respondents with ≥15 completed items. For the main regression analyses examining associations between parental HL and child outcomes, HL was treated as an ordinal continuous variable, and only respondents with sufficient HL data (≥15 completed items) were included.

### 2.3. Sociodemographic and Family Characteristics

Sociodemographic information collected via parental reports included child and parent age and gender, family structure (single-parent household vs. other), and migration background. Parental educational attainment was classified using the Comparative Analysis of Social Mobility in Industrial Nations (CASMIN) index, distinguishing between low, medium, and high education levels [30]. Family socioeconomic position was assessed using the Family Affluence Scale III (FAS III), a validated measure of child and adolescent health research [31,32,33,34]. Residential locations were categorized into urban and rural areas using postal codes.

### 2.4. Psychosocial Context and Child Mental Health Outcomes

Perceived social support was measured using the Multidimensional Scale of Perceived Social Support (MSPSS) proxy-report version [35]. The MSPSS assesses support from family, friends, and significant others, with higher scores indicating greater social support. Child mental health outcomes were assessed using two instruments: emotional and behavioral difficulties were measured using the SDQ parent version [36].

The SDQ comprises five subscales (emotional symptoms, conduct problems, hyperactivity/inattention, peer problems, and prosocial behavior). The total difficulty score was calculated by summing the first four subscales, with scores ranging to from 0 to 40, with higher scores indicating more difficulties.

Psychosomatic complaints were assessed using the Health Behavior in School-aged Children Symptom Checklist (HBSC-SCL) proxy version [37,38]. Parents reported the frequency of eight psychosomatic symptoms (headache, stomachache, backache, feeling low, irritability, nervousness, sleep difficulties, and dizziness) on a five-point scale from “daily” to “not at all.” Symptom items were summed to create a total score (8–40), with higher scores indicating more symptoms.

All measures were treated as proxy-reported indicators of child psychosocial functioning, rather than clinical diagnoses. Instruments were administered in their validated German and Italian versions as previously described [39,40]. Questionnaire language (German vs. Italian) was included as a covariate to account for potential differences in item comprehension and reporting context, reflecting the language actually used during questionnaire completion rather than family or mother tongue. The SDQ was used under a license obtained from the copyright holder, whereas all other instruments were freely available for use in scientific research.

### 2.5. Statistical Analysis

Statistical analyses were performed using IBM SPSS Statistics (version 27). Descriptive statistics were used to characterize the study population. Continuous variables are presented as means and standard deviations, and categorical variables are presented as frequencies and percentages. The internal consistency of the multi-item scales was evaluated using Cronbach’s alpha. Parental HL and proxy-reported child outcomes were analyzed as continuous scores, and categorical classifications were used for descriptive and supplementary analyses. Missing parental HL responses were retained as a separate category to address non-responses.

In addition to the primary missingness definition, a sensitivity analysis used a stricter definition of missing parental HL. Here, parental HL was missing only if participants reached the HL questionnaire block but did not respond to more than one HLS-EU-Q16 item. Participants who ended the survey before the HL section and gave no HLS-EU-Q16 responses were not classified as having missing HL under this strict definition but were considered general survey non-completers with missing HL data. This approach distinguished HL-specific non-responses from questionnaire break-offs, reducing the potential misclassification of missing HL data. To examine whether missing parental HL data were systematically associated with sociodemographic characteristics, logistic regression models were fitted with HL non-response as the dependent variable and parental education, family affluence, migration background, residential area, and parental age as the independent variables. Logistic regression analyses of predictors of missing parental HL were repeated using this definition to assess the robustness of the findings regarding different non-response mechanisms.

Sociodemographic correlates of proxy-reported child psychosocial outcomes were examined using multivariate linear regression. Three models were estimated: (1) emotional and behavioral difficulties, assessed using the SDQ total difficulties score; (2) psychosomatic complaints, assessed using the HBSC-SCL score; and (3) perceived social support, assessed using the MSPSS total score. The models included the following predictors: child age (years), child gender (male vs. female; non-binary cases excluded from gender-stratified analyses), parental age (years), highest parental education (CASMIN categories), family affluence (FAS III categories), migration background (yes vs. no), place of residence (urban vs. rural), and questionnaire language (Italian vs. German). The regression coefficients are reported as unstandardized estimates (β) with 95% confidence intervals, with standardized beta coefficients comparing effect sizes across predictors. Model fit was assessed using the F statistic and adjusted R^2^. Analyses used complete cases for each outcome; denominators varied due to outcome-specific missing data.

Associations between parental HL and proxy-reported child outcomes (SDQ total difficulties score, HBSC-SCL psychosomatic complaint count, and MSPSS perceived social support) were examined using regression models adjusted for sociodemographic covariates. Separate models were estimated for each of the outcomes. Parental HL was entered as an ordinal predictor (per 1-level increase) and models were estimated using complete-case analysis. Accordingly, participants with missing or insufficient HL data were excluded from these models by listwise deletion. The category “missing/insufficient HL” was retained exclusively for descriptive analyses and for the examination of social patterning and mechanisms of non-response. To explore association specificity and assess informant effects, sensitivity analyses were conducted by including perceived social support as a covariate in the models of child mental health outcomes. Covariates were selected based on theoretical relevance and prior evidence and entered simultaneously, regardless of their bivariate association with the outcome. Model diagnostics showed no evidence of multicollinearity (all variance inflation factors [VIF] < 1.3), influential outliers, or major violations of linear regression assumptions.

To facilitate comparisons across psychosocial outcome domains, standardized regression coefficients (standardized β) were derived from fully adjusted linear regression models. Effect sizes were interpreted using conventional benchmarks for standardized coefficients (β ≈ 0.10 small, ≈0.30 moderate, and ≥0.50 large) [41].

All statistical tests were two-sided in nature. Statistical significance was set at *p* < 0.05, with lower thresholds indicating stronger evidence.

### 2.6. Use of Generative Artificial Intelligence (GenAI)

GenAI (ChatGPT 5.2, OpenAI, San Francisco, CA, USA) was used as a language support tool to assist in structuring and refining the manuscript text, including the formulation of the Introduction and Methods sections based on the study protocol and validated instruments. AI was also used for clarity and contextualization to synthesize and cross-reference the existing literature independently identified and selected by the authors. No generative AI was used for data collection, statistical analysis or interpretation of the results. AI tools did not introduce new references or scientific claims; any AI-assisted summaries or rephrasings were cross-checked against the original sources by the authors. All the content was reviewed and approved by the authors. Full responsibility for the scientific content, interpretation, and accuracy of the manuscript rests with the authors, who reviewed and approved the final version in its entirety.

## 3. Results

### 3.1. Study Population, Instrument and Sample Characteristics

The study sample comprised proxy-reported data for 3183 children aged 6–10 years. The internal consistency (Cronbach’s alpha) was 0.926 for the HLS-EU-Q16 scale (valid *n* = 1515), and 0.984 for the 12-item proxy-reported MSPSS total score (valid *n* = 2613). The internal consistency of the SDQ total difficulties score was modest (Cronbach’s α = 0.575, valid *n* = 2471).

The age distribution showed a balanced gender composition. The parents were in mid-adulthood. Most children lived in rural areas without a migration background. The questionnaire was primarily completed in German, reflecting the linguistic composition of the study region. Parental education was skewed toward higher levels, while family socioeconomic position, assessed using the FAS III scale, was mostly medium, with approximately one-quarter of families in the low- and high-affluence categories.

Approximately two-fifths of the parents had adequate HL. Smaller proportions showed problematic or inadequate HL, while over one-third had missing or insufficient HL information. This group comprised parents who terminated the questionnaire before completing the HLS. For analytical transparency, missing and insufficient HL data were categorized separately. The full sample characteristics are presented in Table 1.

### 3.2. Predictors of Missing Parental Health Literacy Data

To assess whether missing or insufficient parental HL was related to sociodemographic or family characteristics, a multivariable logistic regression model was fitted with missing or insufficient HL (HLS-EU-Q16, <15 answered items) as a binary outcome (Table 2).

After mutual adjustment for covariates, neither child age nor gender showed a meaningful association with missing or insufficient parental HL. Parental age was inversely associated with the outcome, indicating a lower likelihood of missing or insufficient HL with increasing age. Parental education is the strongest predictor. Compared with parents with high educational attainment, those with lower or medium education levels showed an increased likelihood of missing or insufficient HL information, following a graded pattern across educational categories. Family affluence was also associated with outcomes. Lower affluence was linked to a higher probability of missing or insufficient parental HL, whereas the association for intermediate affluence was weaker. Migration background and family language were not associated with parental HL non-response after the adjustment. Residential context showed a small but relevant effect, with families in urban areas being less likely to have missing or insufficient parental HL than those in rural areas.

The overall model fit was acceptable. The omnibus test indicated a significant improvement over the null model (χ^2^ = 119.3, df = 10, *p* < 0.001), and the calibration was good (Hosmer–Lemeshow test *p* = 0.502). The model’s explanatory power was limited, with a Nagelkerke R^2^ of 0.052, indicating that the included covariates accounted for only a small proportion of the variance in missing or insufficient parental HL.

In a sensitivity analysis of parents who reached the HL block but provided insufficient responses (1–14 answered items), a logistic regression model was used to test whether very low engagement with the HLS-EU-Q16 (1–5 vs. 6–14 answered items) was associated with sociodemographic or family characteristics (Table S1). None of the predictors were statistically significant. Model calibration was good, but the explanatory power was limited, and the omnibus test showed no improvement over the null model. These findings suggest that there are no systematic sociodemographic determinants of early disengagement from the HL questionnaire in this subgroup.

### 3.3. Descriptive Distribution of Child Mental Health Outcomes

Table 3 summarizes the proxy-reported psychosocial outcomes among children. The mean SDQ total difficulties score was 8.0 (SD 5.7). Based on the cut-offs, 84.0% of the children were in the normal range, 7.3% in the borderline range, and 8.7% in the abnormal range. Psychosomatic complaints were assessed using the HBSC-SCL. The mean HBSC-SCL score was 37 (SD 3.0), ranging from 8 to 40. Perceived social support, measured by proxy-reported MSPSS, was high overall, with a mean score of 5.3 on a 1–7 scale. Three-quarters of the children had high perceived social support, while 10.5% had moderate support and 14.5% had low support.

### 3.4. Sociodemographic Patterning of Psychosocial Outcomes

Table 4 summarizes the multivariable associations between sociodemographic characteristics and proxy-reported psychosocial outcomes among children aged 6–10 years. Across outcomes, the explained variance was low, indicating limited sociodemographic patterning of emotional–behavioral difficulties, psychosomatic complaints and perceived social support. Several consistent associations have been observed.

For emotional and behavioral difficulties (SDQ), male gender, lower parental education, lower family affluence, and migration background were associated with higher difficulty scores. Child age and parental age were not significantly related to SDQ outcomes after adjustment, and associations with residential context and questionnaire language did not attain statistical significance.

For psychosomatic complaints (HBSC-SCL), younger children and higher parental age were associated with higher scores on the HBSC-SCL. Migration background was associated with fewer complaints, whereas parental education, family affluence, residential context, and questionnaire language were not significantly associated with symptom scores. The overall explanatory power remained limited despite its statistical significance.

For perceived social support (MSPSS), higher parental education was associated with greater perceived support, while urban residence and completion of the questionnaire in Italian were associated with lower support. Family affluence showed a borderline positive association with support, whereas child age, child gender, parental age, and migration background were not meaningfully associated.

Although several sociodemographic factors showed significant associations with specific outcomes, the explained variance across models was small, highlighting the limited contribution of sociodemographic characteristics to proxy-reported psychosocial outcomes in this age group.

### 3.5. Associations Between Parental Health Literacy and Proxy-Reported Child Outcomes

Multivariable linear regression models were used to examine the associations between parental HL and proxy-reported psychosocial outcomes in children aged 6–10 years, adjusting for child age and gender, parental age, education, family affluence, migration background, urban residence, and questionnaire language (Table 5). Across all outcomes, parental HL showed significant and consistent associations in all models. Higher parental HL was associated with fewer emotional and behavioral difficulties, more psychosomatic complaints, and higher levels of perceived social support. These associations remained robust after adjusting for socioeconomic and demographic covariates.

Beyond parental HL, emotional and behavioral difficulties were higher among boys and children with a migration background. Psychosomatic complaints were inversely associated with younger child age and higher parental age and were lower among children with a migration background. For perceived social support, higher parental education was associated with greater support, urban residence was associated with lower support scores, whereas Italian questionnaire completion was associated with higher perceived social support compared with German questionnaire completion.

The explanatory power of the models was limited, with adjusted R^2^ values of approximately 0.05 for the SDQ, 0.04 for the HBSC-SCL, and 0.02 for the MSPSS. All models were statistically significant, indicating that the covariates jointly explained a small proportion of the variance in child psychosocial outcomes.

In sensitivity analyses adjusting for perceived social support (MSPSS total score), the associations between parental HL and proxy-reported child mental health outcomes remained significant for both SDQ total difficulties and psychosomatic complaints.

In the MSPSS-adjusted model for SDQ total difficulties, higher parental HL was associated with lower SDQ scores (B = −1.41 per category increase, 95% CI −1.80 to −1.02, *p* < 0.001). Perceived social support was inversely associated with SDQ total difficulties, with higher MSPSS scores linked to lower SDQ scores (B = −0.49, 95% CI −0.64 to −0.34; *p* < 0.001). Male gender was associated with higher SDQ scores (B = 1.31, 95% CI 0.81 to 1.81, *p* < 0.001), as did migration background (B = 1.07, 95% CI 0.32 to 1.83, *p* = 0.006). Child age, parental age, parental education, family affluence, urban residence, and questionnaire language were not significantly associated with the SDQ scores. The adjusted R^2^ was 0.071.

In the MSPSS-adjusted model for psychosomatic complaints, parental HL remained positively associated with symptom counts (B = 0.60, 95% CI: 0.40–0.80, *p* < 0.001). Perceived social support was positively associated with psychosomatic complaints (B = 0.26, 95% CI 0.18–0.34, *p* < 0.001) indicating a non-protective association. Parental age was positively associated with symptom counts (B = 0.05, 95% CI 0.02 to 0.07, *p* < 0.001), whereas migration background was associated with lower symptom counts (B = −0.90, 95% CI −1.29 to −0.51, *p* < 0.001). Child age showed a weak inverse association that did not reach statistical significance, and child gender, parental education, family affluence, urban residence, and questionnaire language were not significantly associated with complaints. The adjusted R^2^ was 0.057.

### 3.6. Comparison of Effect Sizes Across Outcomes (Non-Specificity)

To examine whether parental HL was differentially linked to specific domains of proxy-reported child functioning, standardized effect sizes from fully adjusted regression models were compared across outcomes. The standardized regression coefficients for parental health literacy were similar across the three outcome domains, indicating comparable relative associations when the effects were in standard deviation units. Higher parental HL was linked to fewer emotional and behavioral difficulties (standardized β ≈ −0.16) and fewer psychosomatic complaints (standardized β ≈ −0.13). The association with perceived social support was smaller but directionally consistent, with higher parental HL linked to higher perceived support (standardized β ≈ 0.05). Overall, the similarity in standardized effect sizes across outcome domains indicates that parental HL is not selectively related to a single aspect of child psychosocial functioning. Instead, it represents a broader contextual factor linked to multiple dimensions of proxy-reported child outcomes, even after adjusting for socioeconomic characteristics, including corrected family affluence.

## 4. Discussion

In this population-based study of proxy-reported mental health outcomes in children aged 6–10 years, parental HL was consistently associated with parent-reported emotional difficulties, psychosomatic complaints, and perceived social support. Higher parental HL was related to fewer emotional and behavioral difficulties, but to higher levels of reported psychosomatic complaints, as well as to higher perceived social support, with comparable effect sizes after adjusting for sociodemographic characteristics. The explanatory power of all models was limited, with sociodemographic characteristics accounting for a minimal variance in outcomes. Within this context, parental HL emerged as a consistent correlate across domains, suggesting that it is systematically related to how parents perceive their children’s psychosocial functioning. Although parental HL may influence children’s psychosocial environment and parents’ reporting of child mental health, the modest effect sizes reflect the expected magnitude of a distal determinant in the population models. The consistency across domains supports the view of parental HL as a generalized contextual factor, highlighting the importance of considering informant characteristics when interpreting proxy-reported child mental health data.

### 4.1. Parental Health Literacy and Proxy-Reported Child Mental Health

In this study of children aged 6–10 years, parental HL was associated with proxy-reported psychosocial outcomes across multiple domains. This aligns with the literature showing that higher parental HL is linked to better child health behaviors and psychosocial functioning [13,42,43,44]. However, interpretation requires caution in studies using parent proxy reports, as parental characteristics can shape how child symptoms are perceived and reported [20,45,46,47,48]. Parental HL is intertwined with socioeconomic conditions, education, stress, and mental health, affecting both the children’s psychosocial environment and parental reporting [43,49,50,51]. Parental HL may influence children through effective parenting, appropriate help-seeking, and reduced parental distress [13,42,44,52,53]. Proxy assessments are sensitive to the rater’s mental health and beliefs, with parents with poorer mental health reporting more child problems [46,48]. This explains why parental HL may show broad associations with proxy-reported outcomes, even when actual differences in child psychopathology are modest. In the present study, this pattern did not translate into uniformly lower proxy-reported symptom levels, as higher parental health literacy was associated with higher reporting of psychosomatic complaints, likely reflecting differences in symptom recognition and reporting thresholds rather than poorer child health.

Evidence supports that parental perceptions and reporting thresholds influence the observed associations. First, mental HL determines whether parents recognize symptoms as mental health problems, which varies by parent characteristics, stigma, and familiarity with mental illness [20]. Second, parents’ ratings differ from child self-reports predictably, with larger discrepancies for internal states and psychosocial domains than for observable physical domains [45,47]. In clinical contexts, caregiver fatigue, anxiety, and depression predict the magnitude and direction of caregiver–child discrepancies in symptom ratings, indicating that proxy reports capture caregiver burden and appraisal beyond child status [46]. Third, parental mental health can influence proxy ratings even in long-term follow-up settings, suggesting that informant effects may persist rather than remain situational [48]. Finally, the socio-cultural context shapes parent ratings, with societal and cultural factors affecting the variance in parent-reported child mental health problems across societies [54].

Evidence of psychometric robustness does not eliminate this concern. Measurement invariance studies have shown that the structure of tools like the SDQ may be stable across levels of parental mental health, supporting measurement property comparability [10]. However, invariance does not preclude systematic differences in endorsement levels driven by parental perceptions, symptom salience, or reporting thresholds, particularly in less observable domains [46,47]. Genetically informed literature shows that associations between parental characteristics and child mental health can reflect environmental pathways, shared genetics, and reporting processes, indicating that cross-sectional proxy report associations should not be interpreted as causal child effects [49,51,55].

Our findings align with the literature, suggesting that parental HL is meaningfully related to proxy-reported child psychosocial outcomes, reflecting both family level determinants of child well-being and rater-dependent processes that influence how parents notice, interpret, and report child symptoms [13,20,46,47]. This interpretation supports parental HL as an intervention target, while emphasizing the need for multi-informant assessment strategies when feasible, especially for internalizing and psychosocial domains, where proxy–self discrepancies are larger [45,56,57].

### 4.2. Non-Specificity of Associations Across Outcome Domains

This study found associations of comparable magnitude but differing direction between parental HL and proxy-reported child outcomes across three domains: emotional–behavioral difficulties, psychosomatic complaints, and perceived social support. These associations were comparable, indicating that parental HL had similar effects across outcomes. This non-specific pattern conflicts with narrowly defined child mechanisms and suggests broader contextual processes are at play. Research shows that parental characteristics influence ratings across multiple child outcomes rather than specific domains. Parent–child agreement is lower for internal states and social functioning than for observable behaviors, indicating that parental interpretation plays a key role [45,47].

Comparative studies have shown that discrepancies between parent and child reports are systematically patterned across domains, with similar parental influences on emotional, psychosomatic, and social outcomes [58]. Psychometric evidence indicates that these patterns are not driven by measurement artifacts. Measurement invariance analyses of common instruments, including the SDQ, demonstrate stable factor structures across parental characteristics, supporting scale comparability while allowing systematic differences in response thresholds and symptom salience [10,59]. The associations between parental health literacy and proxy-reported child outcomes suggest that parental HL is not selectively related to specific psychosocial problems. Instead, it acts as a broader contextual factor influencing how parents perceive and report their children’s emotional, psychosomatic, and social functioning. This pattern indicates that parental HL may shape the overall parental appraisal of child well-being rather than reflecting distinct pathways for individual domains. Proxy-reported outcomes should be interpreted cautiously when drawing domain-specific conclusions from them. These findings underline the importance of multi-informant approaches in future research to disentangle child-related phenomena from parental reporting characteristics.

### 4.3. Role of Perceived Social Support and Sensitivity Analyses

To assess robustness, regression models were estimated, including parent-reported perceived social support as a covariate. The associations between parental health literacy and proxy-reported child emotional–behavioral difficulties and psychosomatic complaints were attenuated but remained significant, indicating a partial explanatory overlap with perceived social support. Perceived social support plays a dual role as a family level resource linked to parental HL, well-being, and family functioning. Higher HL is related to better use of social resources and less parental distress, fostering favorable environments for children. Partial reduction after adjustment aligns with partial mediation in the family context. Perceived social support and child outcomes were reported by the same informant, raising concerns about the shared method variance. Research shows that associations between parent-reported predictors and proxy–reported child outcomes are inflated when from a single reporter, particularly for internalizing and psychosomatic domains relying on parental appraisal rather than direct observation [47,60]. Parent proxy ratings reflect parental stress, burden, and interpretive frameworks in addition to child functioning [61,62].

The persistence of associations after adjustment argues against a purely artificial explanation. These findings support the interpretation that parental HL is linked to family resources and reporting tendencies that affect multiple proxy-reported outcomes. Effect estimates from single-informant models reflect both contextual influences and informant-specific variances.

These analyses reinforce the main findings while highlighting the importance of multi-informant approaches in interpreting proxy-reported psychosocial outcomes in children.

### 4.4. Selective Missingness of Parental Health Literacy Data

Analyses of missing parental HL data showed that non-response was socially patterned, not random. Missing HL data were more common among parents with lower education, younger age, and urban residence. This aligns with research showing that individuals with lower education, socioeconomic position, and social disadvantage are more affected by non-response or incomplete HL assessments [63,64,65]. The selective missingness of parental health literacy has key implications for population surveys and epidemiological studies of child mental health. Incomplete parental HL data may bias the estimates of HL levels and associated social gradients. Such bias may result in over- or underestimations of the association between parental HL and proxy-reported child outcomes. Research shows that families at higher psychosocial risk are often underrepresented in complete-case analyses, distorting effect estimates and population inferences [13,64]. Consequently, the public health relevance of parental HL may be misestimated if missing data are not adequately considered.

These findings argue against the assumption that missing HL data are missing at random. While methods such as multiple imputation and inverse probability weighting are recommended for incomplete data, their validity depends on assumptions that may not hold when missingness links to the construct [66,67]. Retaining missing HL as a distinct category can be justified when HL is explanatory, and missingness reflects vulnerability. In this study, treating missing parental HL as a separate category allowed the inclusion of a meaningful subgroup and examination of non-response patterns. This approach supports transparency and analyses that acknowledge the missing-not-at-random mechanisms. The findings highlight the need for reporting strategies that address selective missingness in parental HL, particularly in equity-oriented child health policies.

A sensitivity analysis restricted to parents who reached the HL block of the questionnaire but provided insufficient responses showed no systematic sociodemographic predictors of early disengagement with HL items. This suggests that social patterning mainly affects whether parents provide sufficient HL data rather than how far they proceed once engaged. Accordingly, selective missingness operates primarily at the level of participation in the HL assessment rather than reflecting differential response behavior within the HL block of items.

### 4.5. Methodological Implications for Proxy-Based Child Mental Health Research

These findings highlight the methodological considerations for research using proxy reports of child mental health. They underscore the risk of interpreting proxy reports as direct indicators of child psychopathology without considering parental HL, mental health, or socioeconomic context. Evidence shows that parent proxy reports are influenced by children’s symptoms and parental cognitive, emotional, and contextual factors. Parents with poorer mental health or lower HL report more child difficulties at similar child clinical status levels, showing systematic reporting effects [10,12,46]. This can distort prevalence estimates in epidemiological surveillance when parental distress differs across population subgroups [2,68]. Studies have shown that parent- and child-reported measures identify only partially overlapping risk groups, limiting proxy-only screening validity [57].

Measurement invariance studies indicate that instruments such as the SDQ maintain a stable psychometric structure across parental characteristics, supporting comparability at the scale level [10]. However, invariance does not rule out systematic differences in endorsement levels due to parental appraisal, symptom salience, or reporting thresholds. Thus, observed group differences may partly reflect informant composition rather than true differences in child mental health [2]. These issues are particularly relevant to intervention studies targeting parental HL. Improvements in parental knowledge may change how parents perceive and report child symptoms, even with modest changes in child mental health. In primary care, pediatricians and general practitioners are well positioned to improve parental HL through repeated brief communication strategies, including plain language, key messages, and teach-back during routine consultations. While such interventions benefit parenting practices and help-seeking [13,19,68], their effects on proxy-reported child outcomes should be interpreted cautiously and corroborated using additional informants or assessment methods. The findings emphasize the need for multi-informant designs and consideration of informant characteristics in proxy-based child mental health research, especially in population surveillance and intervention evaluation [55,56].

### 4.6. Strengths and Limitations

This study had several strengths. First, it used a large population-based sample of children aged 6–10 years, enhancing generalizability to community settings. Second, validated instruments captured different domains of child mental health and psychosocial functioning, allowing for comparable assessments across outcomes. Third, the analyses included adjustments for sociodemographic and family related covariates, reducing potential confounding. Fourth, this study examined patterns of missing parental HL data and addressed them analytically through sensitivity analyses. These features strengthen the internal consistency and methodological transparency of the study.

The interpretation of the findings should consider the characteristics and scope of the measurement instruments used. All outcomes were assessed using established questionnaires that operationalize specific constructs through predefined item sets. Consequently, the observed associations reflect relationships within these operational definitions and may differ if alternative instruments or broader measures were applied. For example, perceived social support was measured using the Multidimensional Scale of Perceived Social Support, which focuses on support from family, friends, and significant others, but does not capture other potentially relevant sources such as community organizations, religious networks, or online social environments. Similar instrument-specific constraints apply to the SDQ and HBSC-SCL, which emphasize particular symptom domains and reporting formats. These measurement characteristics may influence the magnitude and direction of observed associations and should be considered when generalizing findings beyond the specific instruments employed.

This study has several limitations. The cross-sectional design precludes causal inference and temporal relationships between parental HL, perception, and child psychosocial outcomes. The absence of child self-reports or clinical assessments limits the distinction between true child psychopathology and perception-related effects. The internal consistency of the SDQ total difficulties score was modest in this sample, reflecting the heterogeneous content of the scale and the proxy-report format in younger children; this may have attenuated observed associations and warrants cautious interpretation of SDQ-based findings.

In addition, the analyses did not account for family composition variables such as the number of children in the household or the ages of siblings. These factors may influence parental stress, attention allocation, and comparative appraisal processes, potentially affecting proxy reporting of child psychosocial outcomes. As the present study focused on informant characteristics and reporting-related mechanisms rather than family-system dynamics, these variables were not included.

Finally, although broad covariates were included, residual confounding by unmeasured parental or contextual factors such as mental health or parenting styles cannot be ruled out.

## 5. Conclusions

In this population-based study of children aged 6–10 years, parental HL was consistently associated with proxy-reported children’s emotional and behavioral difficulties, psychosomatic complaints, and perceived social support, with outcome-specific directions of association. The similarity of effect sizes across outcome domains and the limited explanatory power of sociodemographic covariates suggest that parental HL functions as a broad contextual and reporting-related factor rather than as a determinant of specific aspects of child psychosocial functioning. These findings highlight the importance of considering informant characteristics when interpreting proxy-reported child mental health data, particularly in epidemiological surveys and screening. While parental HL remains a relevant target for family- and population-level interventions, future research should prioritize multi-informant designs and longitudinal approaches to better disentangle the true child effects from parental perception and reporting processes.

## Figures and Tables

**Table 1:** Sample characteristics of children aged 6–10 years (proxy-reported).

Characteristic	Total (*n* = 3183)
Child age, years, mean (SD)	8.0 (1.40)
Child gender, *n* (%)	
Female	1581 (49.7)
Male	1601 (50.3)
Diverse	1 (<0.1)
Parental age, years, mean (SD)	40.8 (5.40)
Migration background, *n* (%)	
No	2747 (86.6)
Yes	424 (13.4)
Residence, *n* (%)	
Rural	2232 (70.1)
Urban	951 (29.9)
Questionnaire language, *n* (%)	
German	2540 (79.8)
Italian	643 (20.2)
Parental education (CASMIN), *n* (%)	
Low	429 (13.6)
Medium	1139 (36.2)
High	1578 (50.2)
Family Affluence Scale III, *n* (%)	
Low	763 (24.5)
Medium	1619 (52.0)
High	730 (23.5)
Parental health literacy category, *n* (%) ^1^	
Inadequate	214 (6.7)
Problematic	541 (17.0)
Adequate	1283 (40.3)
Missing/insufficient health literacy items	1145 (36.0)

Values are mean (SD) or *n* (%); percentages are calculated based on valid responses for each variable and may not sum to 100% because of missing data. ^1^ Cases of parental health literacy with missing or insufficient health literacy data were retained as a separate category including *n* = 254 missing and *n* = 891 insufficient. Abbreviations: SD, standard deviation; CASMIN, Comparative Analysis of Social Mobility in Industrial Nations.

**Table 2:** Multivariable logistic regression analysis of sociodemographic predictors of missing or insufficient parental health literacy (HLS-EU-Q16; <15 answered items), *n* = 3075.

Predictor	Reference Category	OR	95% CI	*p*-Value
Child age (years)	per year	1.03	0.98–1.09	0.278
Child gender (male vs. female)	female	0.96	0.83–1.12	0.635
Parental age (years)	per category increase	0.98	0.96–0.99	0.002
Parental education (CASMIN)	high			<0.001
Low vs. High	high	2.56	1.78–3.69	<0.001
Medium vs. High	high	1.93	1.59–2.35	<0.001
Family affluence category (FAS III)	high			0.002
Low vs. High	high	1.49	1.19–1.87	<0.001
Medium vs. High	high	1.21	0.99–1.47	0.059
Migration background (yes vs. no)	no	1.05	0.84–1.33	0.659
Urban residence (urban vs. rural)	rural	0.83	0.68–1.00	0.049
Questionnaire language (Italian vs. German)	German	0.95	0.77–1.19	0.671

Odds ratios (ORs) represent adjusted estimates from multivariable logistic regression models. ORs > 1 indicate a higher likelihood of missing or insufficient parental health literacy, defined as fewer than 15 valid responses on the HLS-EU-Q16. The dependent variable was coded as 1 for missing or insufficient health literacy and 0 for valid health literacy (≥15 items). All predictors were simultaneously entered into the model. Abbreviations: CASMIN, Comparative Analysis of Social Mobility in Industrial Nations; FAS III, Family Affluence Scale III.

**Table 3:** Descriptive distribution of child psychosocial outcomes (children aged 6–10 years, proxy-reported).

Outcome	*n*	Score ^1,2^/Count ^3^		Categories, *n* (%)		
		Mean (SD)	Min–Max	Normal ^1^/ Low ^2^	Borderline ^1^/ Moderate ^2^	Abnormal ^1^/ High ^2^
Emotional behavioral difficulties (SDQ)	2471	8.0 (5.72)	0–37	2076 (84.0)	181 (7.3)	214 (8.7)
Psychosomatic complaints (HBSC-SCL)	2496	37.0 (2.96)	8–40	—	—	—
Perceived social support (MSPSS)	2613	5.3 (1.79)	1–7	380 (14.5)	274 (10.5)	1959 (75.0)

^1^ SDQ total difficulties score, categories (normal, borderline, abnormal); ^2^ MSPSS total score, categories (low, moderate, high); ^3^ HBSC-SCL scores. Values were based on proxy reports from parents or legal guardians. Percentages refer to valid cases for each variable; denominators vary because of outcome-specific missing data. Abbreviations: SD, standard deviation; SDQ, Strengths and Difficulties Questionnaire; HBSC-SCL, Health Behavior in School-aged Children Symptom Checklist; MSPSS, Multidimensional Scale of Perceived Social Support.

**Table 4:** Multivariable linear regression analyses of psychosocial outcomes in children.

Predictor	SDQ Total Difficulties Score		HBSC-SCL Psychosomatic Complaints		MSPSS Social Support Score	
	B (95% CI)	*p*	B (95% CI)	*p*	B (95% CI)	*p*
Child age (years)	−0.04 (−0.21; 0.13)	0.653	−0.13 (−0.22; −0.04)	0.004	−0.02 (−0.07; 0.03)	0.419
Child gender (male vs. female)	−1.25 (−1.70; −0.80)	<0.001	0.08 (−0.15; 0.32)	0.490	0.01 (−0.12; 0.15)	0.848
Parental age (years)	−0.03 (−0.08; 0.02)	0.184	0.05 (0.03; 0.08)	<0.001	0.00 (−0.01; 0.02)	0.910
Parental education (CASMIN, higher = higher category)	−0.85 (−1.31; −0.39)	<0.001	0.03 (−0.21; 0.27)	0.817	0.45 (0.31; 0.58)	<0.001
Family affluence (FAS III, higher = higher category)	−0.40 (−0.73; −0.06)	0.020	0.13 (−0.04; 0.31)	0.131	0.10 (−0.00; 0.20)	0.054
Migration background (yes vs. no)	0.98 (0.29; 1.68)	0.006	−0.90 (−1.26; −0.54)	<0.001	−0.10 (−0.31; 0.11)	0.359
Residence (urban vs. rural)	0.51 (−0.05; 1.06)	0.074	0.05 (−0.24; 0.34)	0.733	−0.18 (−0.35; −0.01)	0.039
Questionnaire language (Italian vs. German)	0.54 (−0.12; 1.20)	0.107	−0.15 (−0.48; 0.19)	0.399	0.21 (0.01; 0.41)	0.037
*n*	2429		2455		2570	
Adjusted R^2^	0.026		0.017		0.020	
Overall model F (df1, df2), *p*	F(8, 2420)	<0.001	F(8, 2446)	<0.001	F(8, 2561)	<0.001

Values are unstandardized regression coefficients (B) with 95% confidence intervals derived from multivariable linear regression models estimated using the enter method. Child gender was modeled as a binary contrast (male vs. female); the single case reporting a diverse gender was excluded from this contrast and treated as missing. Higher SDQ and HBSC-SCL scores indicate greater emotional and behavioral difficulties or psychosomatic symptom burden, whereas higher MSPSS scores indicate greater perceived social support. All models were estimated using complete-case analysis. Sample sizes varied across outcomes because of outcome-specific missing data and listwise deletion within each model. Abbreviations: SDQ, Strengths and Difficulties Questionnaire; HBSC-SCL, Health Behavior in School-aged Children Symptom Checklist; MSPSS, Multidimensional Scale of Perceived Social Support; CI, confidence interval.

**Table 5:** Multivariable linear regression analyses of the association between parental health literacy and proxy-reported child outcomes (children aged 6–10 years).

Predictor	SDQ ^3^ Total Difficulties Score		HBSC-SCL ^3^ Psychosomatic Complaints		MSPSS ^3^ Perceived Social Support	
	B (95% CI)	*p*	B (95% CI)	*p*	B (95% CI)	*p*
Parental health literacy (per 1-level increase) ^1^	−1.40 (−1.79; −1.01)	<0.001	0.61 (0.40; 0.81)	<0.001	0.14 (0.02; 0.26)	0.018
Child age (years)	−0.04 (−0.22; 0.14)	0.699	−0.11 (−0.20; −0.01)	0.035	−0.05 (−0.11; 0.00)	0.068
Child gender (male vs. female) ^2^	−1.26 (−1.75; −0.76)	<0.001	0.09 (−0.176; 0.35)	0.510	0.08 (−0.072; 0.23)	0.309
Parental age (years)	−0.03 (−0.08; 0.02)	0.312	0.05 (0.025; 0.079)	<0.001	0.01 (−0.01; 0.02)	0.456
Parental education (per 1-level increase)	−0.41 (−0.94; 0.13)	0.138	−0.04 (−0.32; 0.26)	0.815	0.31 (0.15; 0.47)	<0.001
Family affluence (per 1-level increase)	−0.31 (−0.68; 0.06)	0.105	0.16 (−0.04; 0.36)	0.121	0.10 (−0.010; 0.21)	0.074
Migration background (yes vs. no)	1.25 (0.50; 1.00)	0.001	−1.02 (−1.42; −0.62)	<0.001	−0.19 (−0.41; 0.03)	0.089
Urban residence (vs rural)	0.49 (−0.11; 1.09)	0.108	0.04 (−0.28; 0.36)	0.812	−0.21 (−0.40; −0.03)	0.020
Questionnaire language (Italian vs. German)	0.58 (−0.11; 1.28)	0.101	−0.18 (−0.55; 0.19)	0.333	0.22 (0.01; 0.430)	0.038
*n*	1956		1982		2056	
Adjusted R^2^	0.049		0.036		0.017	
Overall model F (df1, df2), *p*	F(9, 1946)	<0.001	F(9, 1972)	<0.001	F(9, 2046)	<0.001

Values are unstandardized regression coefficients (B) with 95% confidence intervals (CI) from multivariable linear regression models (enter method). ^1^ Parental health literacy was entered as an ordinal continuous predictor (per 1-level increase) in the regression models. Analyses were conducted using complete cases; participants with missing or insufficient health literacy data (<15 completed HLS-EU-Q16 items) were excluded from these models by listwise deletion. ^2^ Child gender was modeled as male vs. female; the single “diverse” case was excluded from this binary contrast (handled as missing in SPSS). ^3^ Higher SDQ and HBSC-SCL scores indicate greater difficulties or symptom burden, whereas higher MSPSS scores indicate greater perceived social support. Sample sizes differed across outcomes due to outcome-specific missingness and listwise deletion. Abbreviations: SDQ, Strengths and Difficulties Questionnaire; HBSC-SCL, Health Behavior in School-aged Children Symptom Checklist; MSPSS, Multidimensional Scale of Perceived Social Support; CI, confidence interval.

## Data Availability

The data presented in this study are available from the corresponding author upon reasonable request.

## Supplementary Materials

The following supporting information can be downloaded at: https://www.mdpi.com/article/10.3390/children13020253/s1, Table S1: Predictors of very low engagement with the HLS-EU-Q16 among parents with insufficient response completeness (1–14 answered items).

## Abbreviations

The following abbreviations are used in this manuscript:

CASMIN	Comparative Analysis of Social Mobility in Industrial Nations
CI	Confidence interval
COVID-19	Corona virus disease 2019
FAS III	Family Affluence Scale III
HBSC-SCL	Health Behavior in School-aged Children Symptom Checklist
HL	Health literacy
HLS-EU-Q16	European Health Literacy Survey Questionnaire 16 Items
MSPSS	Multidimensional Scale of Perceived Social Support
OR	Odds ratio
SD	Standard deviation
SDQ	Strengths and Difficulties Questionnaire
VIF	Variance inflation factor
