# Supplementary material for: Parental Health Literacy as a Contextual Factor in Proxy-Reported Child Mental Health: A Population-Based Study of Children Aged 6–10 Years
Source: Children (Basel). 2026 Feb 11;13(2):253. doi: 10.3390/children13020253 (PMC12939823; doi:10.3390/children13020253)
Supplement: Supplementary file 1 [file children-13-00253-s001.zip › children-4146348-supplementary.pdf]

Supplementary Materials

Table S1. Predictors of very low engagement with the HLS-EU-Q16 among parents with insufficient response completeness (1–14 answered items).

Predictor	Reference category	OR	95% CI	*p*-value
Child age (years)	per year	1.06	0.82–1.37	0.656
Child gender (male)	Female	1.04	0.54–2.03	0.905
Parental age (years)	per category increase	0.96	0.90–1.03	0.251
Parental education	High			0.321
Low vs High	High	2.24	0.78–6.47	0.135
Medium vs High	High	1.06	0.47–2.38	0.887
Family affluence category	High			0.870
Low vs High	High	1.29	0.43–3.84	0.646
Medium vs High	High	1.30	0.48–3.57	0.606
Migration background	No	1.47	0.47–4.60	0.512
Urban residence	Rural	1.49	0.64–3.47	0.354
Questionnaire language	German	1.27	0.47–3.42	0.632

This supplementary analysis was restricted to parents who reached the health literacy block but provided an insufficient number of responses for score calculation (1–14 of 16 HLS-EU-Q16 items). The outcome contrasts very low engagement (1–5 answered items) with higher engagement within the insufficient range (6–14 answered items). Odds ratios (ORs) represent adjusted estimates from a multivariable logistic regression model restricted to parents who reached the health literacy block but provided an insufficient number of responses for score calculation (1–14 of 16 HLS-EU-Q16 items; n = 869). The dependent variable contrasted very low questionnaire engagement (1–5 answered items; n = 37) with higher response completeness within the insufficient range (6–14 answered items; n = 832). All predictors were entered simultaneously. Model calibration was good (Hosmer–Lemeshow test p = 0.945). Explanatory power was low (Nagelkerke R^2 = 0.023), and the omnibus test of model coefficients was not significant (χ^2 = 5.84, df = 10, p = 0.829). Abbreviations: CASMIN, Comparative Analysis of Social Mobility in Industrial Nations; FAS III, Family Affluence Scale III; HLS-EU-Q16, European Health Literacy Survey Questionnaire (16-item version).
